# Next-generation sequencing yields the complete mitogenome of wattled crane (*Bugeranus carunculatus*)

**DOI:** 10.1080/23802359.2018.1481791

**Published:** 2018-06-29

**Authors:** Rong Hua, Duoying Cui, Jia Liu, Yuyan You, Ting Jia

**Affiliations:** Beijing Key Laboratory of Captive Wildlife Technologies, Beijing Zoo, Beijing, China

**Keywords:** Complete mitogenome, wattled crane, next-generation sequencing

## Abstract

In this study, the complete mitogenome sequence of wattled crane (*Bugeranus carunculatus*) has been decoded by next-generation sequencing and genome assembly. The assembled mitogenome, consisting of 16,679 bp, has unique 14 protein-coding genes (PCGs), 22 transfer RNAs and 2 ribosomal RNAs genes. The complete mitogenome provides essential and important DNA molecular data for further phylogenetic and evolutionary analysis for wattled crane phylogeny.

Cranes inhabit open habitats and are among the world’s most threatened bird families. The wattled crane (*Bugeranus carunculatus*) is a large bird found in Africa, which populations are widely distributed in the southern Africa region, particularly in Angola, Botswana, Democratic Republic of Congo, Malawi, Mozambique, Namibia, Zambia and South Africa. Globally, wattled crane is listed as vulnerable and endangered species, under the International Union of Conservation for Nature (IUCN) Red Data List (Daso et al. 2015; Fakarayi et al. 2016).

Peripheral blood of the wattled crane (♂) were collected from Beijing Zoo in Beijing, China. The specimens were kept in the laboratory at −80 °C, total genomic DNA was extracted using a DNA extract kit following the previously reports (Bai et al. 2010; Günal et al. 2018). And we used next-generation sequencing to perform low coverage whole genome sequencing. Initially, the raw next-generation sequencing reads generated from Illumina HiSeq PE150 (Illumina, San Diego, CA, USA). 40,312,606 clean reads were de novo assembly by using commercial software (Geneious V9, Auckland, New Zealand) to produce a single, circular form of complete mitogenom. The total length of its mitogenome was 16,679 bp, with a genome size similar to other research (NC_020571.1), but more details on that study. Compared with the current mitogenome sequences of wattled crane, there were two bases insertion, and 125 bases substitution in the wattled crane mitogenome we sequenced. The accurate annotated mitochondrial genome sequence was submitted to GeneBank with accession number MH041486. The complete mitogenome of wattled crane crane was 16,679 bp in size and its overall base composition is 31.6% for A, 31.0% for C, 13.4% for G and 24.0% for T, and have GC content of 44.4%.

The protein coding, rRNA and tRNA genes of wattled crane mitogenome were predicted by using DOGMA, ARWEN and MITOS tools and manually inspected. The complete mitogenome of wattled crane includes unique 14 protein-coding genes (PCGs), 22 transfer RNA genes and two ribosomal RNA genes, all details were listed in GeneBank (MH041486) and shown in Figure 1.

**Figure 1.**
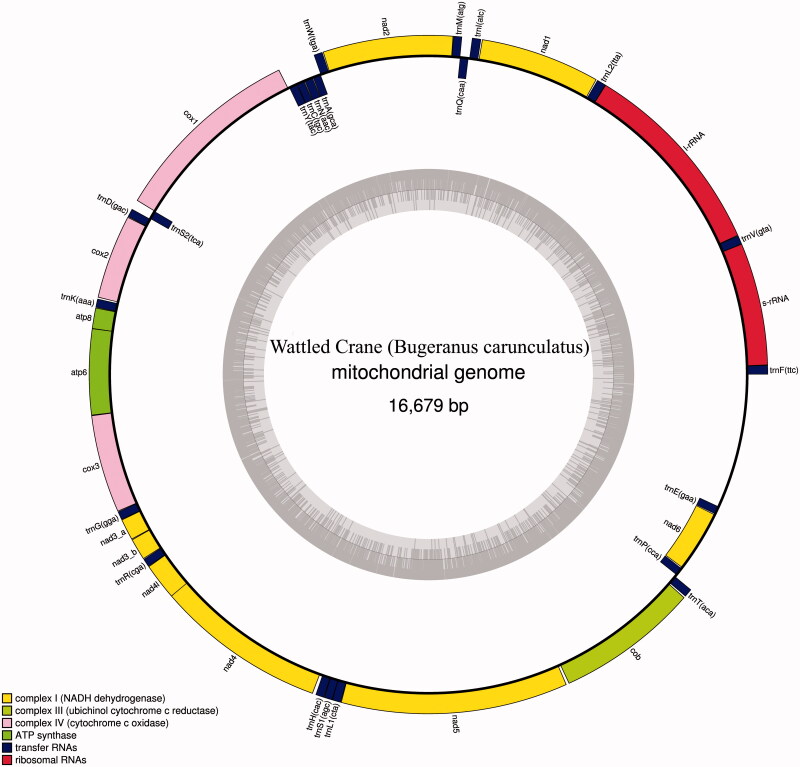
The mitochondrial genome of wattled crane *(Bugeranus carunculatus)*. Gene order and positions are shown, including the putative control region.

To further validate the new sequences, we used all of mitochondrial genome sequences published in GeneBank of other crane species to construct the phylogenetic tree. These species were as follows: *Struthio camelus, Grus japonensis, Grus Americana, Grus grus, Grus monacha, Grus nigricollis, Grus carunculatus, Anthropoides paradiseus, Grus vipio, Grus rubicunda, Grus Antigone, Grus Canadensis and Grus leucogeranus*. We used MEGA6 to produce the phylogenetic tree based on the maximum-likelihood method (Figure 2). The phylogenetic analysis results support that the mitochondrial DNAs of wattled crane are more closely related to other species of crane genus.

**Figure 2.**
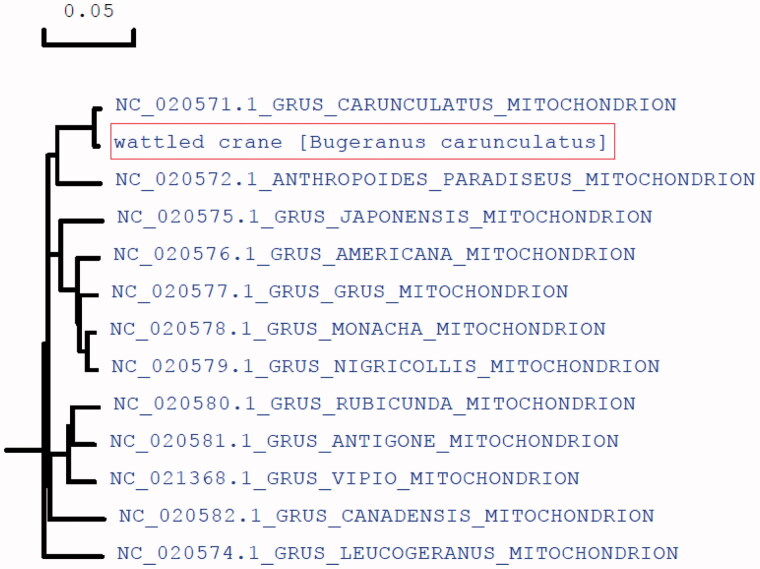
Molecular phylogeny of wattled crane (*Bugeranus carunculatus*) and related species in Crane based on complete mitogenome. The complete mitogenomes are downloaded from GeneBank and the phylogenetic tree is constructed by maximum-likelihood method with 500 bootstrap replicates. The gene’s accession number for tree construction is listed in front of the species name.
